# Pathogenesis of the 1918 Pandemic Influenza Virus

**DOI:** 10.1371/journal.ppat.1001218

**Published:** 2011-01-27

**Authors:** Tokiko Watanabe, Yoshihiro Kawaoka

**Affiliations:** 1 Influenza Research Institute, Department of Pathobiological Sciences, School of Veterinary Medicine, University of Wisconsin-Madison, Madison, Wisconsin, United States of America; 2 ERATO Infection-Induced Host Responses Project, Japan Science and Technology Agency, Saitama, Japan; 3 Division of Zoonosis, Department of Microbiology and Infectious Diseases, Graduate School of Medicine, Kobe University, Kobe, Japan; 4 Division of Virology, Department of Microbiology and Immunology, Institute of Medical Science, University of Tokyo, Tokyo, Japan; 5 International Research Center for Infectious Diseases, Institute of Medical Science, University of Tokyo, Tokyo, Japan; University of California San Francisco, United States of America

At the height of World War I, the human population was assaulted by a powerful, but very small, foreign agent that rapidly appeared seemingly from nowhere. Ultimately identified as the “Spanish flu”, this agent wreaked havoc on anyone in its path. Prostrating vast numbers of victims worldwide with severe pneumonia, which often progressed to a fatal outcome, the “Spanish flu” caused an estimated 20–50 million deaths worldwide [1]. The resultant 1918 pandemic was one of the most formidable foes faced by humankind. In this brief review, we discuss some recent insights into the pathogenicity of its causative agent, the 1918 pandemic influenza virus.

## The “Spanish” Influenza Pandemic of 1918–1919

In early March of 1918, several severe cases of influenza were reported in the United States. This would mark the beginning of the first wave of the “Spanish” influenza pandemic (reviewed in [1]). As massive numbers of US military troops were deployed in Europe, the virus spread too, leading to outbreaks throughout the United States, Europe, and possibly Asia. Spain was neutral during the war and its press was therefore uncensored. Accordingly, the Spanish newspapers were filled with reports of the disease, especially when the king became ill. It is believed that these published accounts of the spread of the disease explain why the virus became known as the “Spanish” influenza. During the first wave of this, like other, pandemics, mortality rates were in the normal range, though morbidity rates were high. However, while the first wave killed relatively few, by the time the next wave came, in the fall of 1918, the virus had undergone numerous passages through humans, had changed dramatically, and was now lethal. Together with a third wave, which occurred in the winter of 1918–1919, approximately 30% of the world's population (500 million people) is thought to have been clinically affected by the 1918 pandemic [1]. Unexpectedly, the 1918 pandemic attacked particularly young adults, who usually have a low death rate during influenza epidemics. As a result, influenza and pneumonia death rates for 15- to 34-year-olds were more than 20 times higher in 1918 than in the previous year (the mortality rate associated with the 1918 virus was more than 2.5% among infected persons compared with less than 0.1% in other influenza epidemics; [1]). Most deaths were caused from secondary bacterial pneumonia due to a lack of antibiotics [1]. The 1918 pandemic virus, however, also killed quickly and directly with a violent viral pneumonia, often with either massive acute pulmonary hemorrhage or pulmonary edema. The disease course was frequently less than 5 days [1].

## Efforts to Unmask the Deadly 1918 Pandemic Virus

The identity of the 1918 pandemic virus remained a mystery in the absence of technology to isolate viruses. By the early 1930s, American and British researchers succeeded in establishing a system to isolate swine and human influenza viruses [2], [3]. With this “new” technique in hand, researchers in the 1950s tried to isolate the 1918 pandemic virus from victims of the pandemic who were buried in permafrost graves in Alaska. However, they were unable to recover virus from the available specimens.

In the late 1990s, the development of molecular biotechnology brought about a breakthrough. Taubenberger's group at the US Armed Forces Institute of Pathology succeeded in isolating viral genome RNA fragments from formalin-fixed, paraffin-embedded autopsy tissues of American army soldiers who had died in the 1918 pandemic [4]. They also isolated viral RNAs from an Alaskan Inuit woman who had been buried in permafrost in Alaska [5]. With these samples, they were able to determine the coding sequences of all eight RNA segments of the 1918 pandemic virus [4]–[10]. This 10-year endeavor finally led them to identify the pathogenic agent that had caused the great 1918 pandemic: that is, influenza A virus subtype H1N1. Further sequencing analyses suggested that the 1918 virus may be of avian origin and transmitted from birds to humans directly or indirectly, although this is remains controversial. In the process of adaptation to humans through mutations, the 1918 virus is thought to have acquired its high virulence.

## The Resurrection of the “Spanish” Influenza Virus

Although the complete sequences of the viral RNAs of the 1918 pandemic virus have been determined, the viral genome does not contain any motifs known to be associated with high virulence [5]. Therefore, to understand the extraordinary virulence of the 1918 pandemic virus, it was important to re-create the virus and examine its pathogenicity in animals. The recent technological advancement of reverse genetics, which allows the generation of infectious influenza viruses entirely from cDNAs [11], made possible the re-creation of the 1918 pandemic virus. Tumpery's group at the US Centers for Disease Control and Prevention [12] and we [13] succeeded in rescuing viruses bearing all eight RNA segments of the 1918 virus by using reverse genetics. Now that we had all of the materials required, we could study the molecular properties associated with the unusual virulence of the 1918 pandemic virus.

## Aberrant Immune Responses in Animals Infected with the Reconstructed 1918 Virus

**Figure 1:**
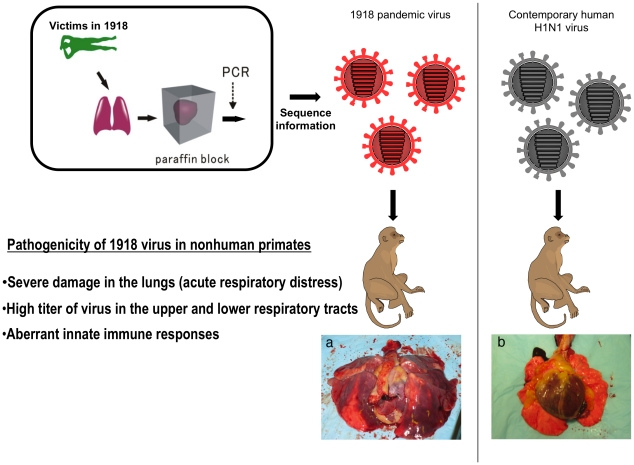
Pathogenicity of the 1918 pandemic influenza virus in non-human primates. Taubenberger's group isolated viral genome RNA fragments from formalin-fixed, paraffin-embedded autopsy tissue from victims of the 1918 pandemic and determined the coding sequences of all eight RNA segments of the 1918 pandemic virus. Based on these sequences, a virus bearing all eight RNA segments of the 1918 virus was generated by reverse genetics and its pathogenicity was determined in a non-human primate model. The 1918 virus caused a highly pathogenic respiratory infection in non-human primates that culminated in acute respiratory distress and a fatal outcome. The infected animals mounted an immune response, characterized by dysregulation of the antiviral response, indicating that atypical host innate immune responses may contribute to lethality. The infection of nonhuman primates with the 1918 pandemic virus caused severe damage in the lungs (a), whereas no appreciable lesions were observed in the lungs of animals infected with a contemporary human H1N1 virus (b).

To derive a molecular mechanism of pathogenicity for the 1918 virus, the reconstructed 1918 virus was characterized in mouse and non-human primate models [12]–[14]. In the infected animals, the 1918 virus replicated to high levels and spread rapidly throughout the respiratory tract. Severe damage, including extensive edema and hemorrhagic exudates, was observed in the lungs of the virus-infected animals [13], as reported for patients who succumbed to the “Spanish” influenza, leading to acute respiratory distress and death (Figure 1). Host responses to the 1918 virus were also investigated by using microarray technology [13], [14]. Strikingly, infection of non-human primates with the 1918 virus triggered high, sustained expression of genes involved in innate immune responses, such as proinflammatory cytokines and chemokines [13]. On the other hand, the virus induced fewer type I interferon genes [13], likely leading to enhanced viral replication. Such uncontrolled innate immune responses have also been observed in mice inoculated with the 1918 virus [14] and in hosts infected with avian H5N1 influenza viruses. These findings suggest that strong immune responses are a hallmark of highly pathogenic influenza virus infections.

## Determinants of the High Virulence of the 1918 Virus in Animals

**Figure 2:**
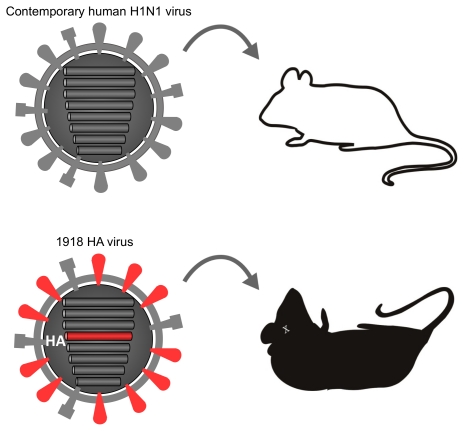
The role of the HA gene in the pathogenicity of the 1918 virus in mice. Mice infected with a contemporary human H1N1 influenza virus showed no symptoms. In contrast, a virus possessing the HA gene derived from the 1918 virus in the genetic background of the contemporary human H1N1 virus was lethal to mice.

Which viral factor(s) contribute to the extraordinary virulence of the 1918 virus? One of the key players is hemagglutinin (HA), which is the viral surface glycoprotein that has two functions in the early stage of virus replication: receptor binding and membrane fusion. The HAs of highly pathogenic avian influenza viruses play a critical role in virulence as they typically contain a specific motif (i.e., a series of basic amino acids at the cleavage site [15], [16]) that contributes to pathogenicity. This motif, however, is not found in the 1918 HA sequence. Nonetheless, we and others demonstrated that a reassortant virus possessing the 1918 HA gene in the genetic background of a contemporary human virus replicated to a high titer in the lungs and caused severe lung damage accompanied by a significant influx of neutrophils and alveolar macrophages into the lung [17], [18](Figure 2). Similar findings were observed with the authentic 1918 virus [19] with severe morbidity and eventual death. These results suggest a critical role for the HA gene in the pathogenicity of the 1918 virus . The region(s) of HA responsible for high virulence has not yet been identified; however, Taubenberger's group has demonstrated that HA receptor binding specificity plays a role in pathogenesis in mice, although their results also suggest the presence of additional virulence determinant(s) [20]. The importance of the 1918 HA, especially two amino acids that are responsible for receptor binding specificity [21] and efficient virus transmission, has also been demonstrated in ferrets [22], [23].

**Figure 3:**
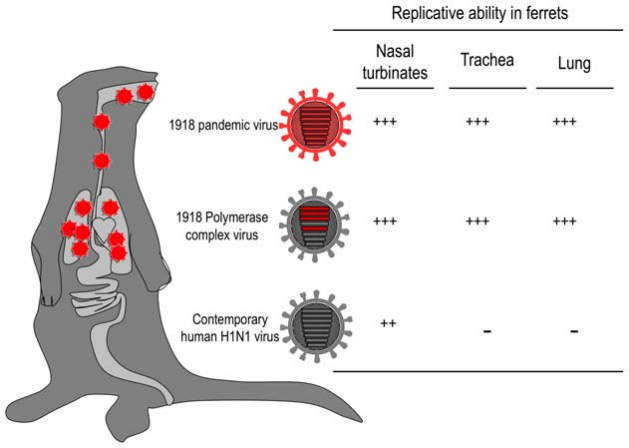
The role of the viral RNA polymerase complex in the replication properties of the 1918 virus in ferrets. The 1918 wild-type virus and a virus possessing the 1918 viral RNA polymerase complex genes (i.e., PA, PB1, PB2, and NP) replicated efficiently in both the upper and lower respiratory tracts of ferrets, whereas the replication of the contemporary human H1N1 virus was restricted to the upper respiratory tract, suggesting a role for the viral RNA polymerase complex in the optimal growth of the 1918 virus in the lower respiratory tract of ferrets. +++, high replicative ability; ++, moderate replicative ability; −, no virus detected.

The other important player in the virulence of the 1918 virus is the viral RNA polymerase complex. A unique feature of the 1918 pandemic was that many people died from viral pneumonia; human influenza viruses typically replicate poorly in the lungs of infected individuals and rarely cause fatal viral pneumonia. We reported efficient replication of the 1918 virus in the lungs of infected ferrets and non-human primates, which led to viral pneumonia [13], [24]. In contrast, a contemporary human H1N1 virus was not detected in the lungs of infected animals, although it replicated in the nasal turbinates. Hence, we assume that the ability of the 1918 virus to grow in the lungs is associated with its high virulence in humans. We also found that high virus titers in the lungs of infected ferrets were dependent on the 1918 polymerase and NP genes (Figure 3) [24]. The importance of the polymerase genes in pathogenicity in mice [25] and transmission in ferrets [22] was also shown. These findings strongly implicate the viral RNA polymerase complex in the efficient spread of virus to the lower respiratory tract and suggest it may be required, together with a specific HA, to induce the type of fatal pneumonia encountered during the 1918–1919 pandemic.

Other viral factors may also be involved in the pathogenicity of the 1918 pandemic virus, such as PB1-F2, NA, and NS1 [25]–[28]. PB1-F2, a proapoptotic viral protein, requires only a single amino acid change at position 66 to increase the pathogenicity of the 1918 virus [26]. Expression of the 1918 PB1-F2 promotes lung pathology in primary viral infection and secondary bacterial infection [27]. A potential role for the NA gene in optimal replication and virulence of the 1918 virus has also been suggested [25]. For NS1, an antagonist of cellular innate immune defenses, the 1918 protein is better able to block the expression of IFN-regulated genes than is the NS1 protein of other human influenza viruses [28]. Moreover, the C-terminal of NS1 is known to contribute to enhanced virulence and pathogenesis by a mechanism distinct from its IFN-antagonistic properties [29]. Despite these important functions of NS1, substitution of the NS gene of the 1918 virus for that of a parental virus caused attenuation of the parental virus in mice [10], [25].

## Concluding Remarks

The first influenza pandemic of the 21st century was caused by a novel swine-origin H1N1 influenza virus that emerged in early 2009. This virus is substantially less virulent than the 1918 influenza virus, but it has the potential to acquire amino acid changes in its viral proteins that would increase its pathogenicity. To prepare for such events and future pandemics, we need to understand the molecular basis of the high-virulence phenotype of the 1918 pandemic virus to help identify virulence factors in other emerging pandemic viruses. Additionally, the fact that more than 97% of the people infected with the 1918 virus survived raises the intriguing possibility of some contribution of host genetics to the consequences of influenza (i.e., survival or death). Thus, it is also important to explore host factors that are involved in resistance or susceptibility to influenza virus infection. Such information could accelerate the development of new antiviral drugs for prophylaxis and treatment, which are urgently needed given the obstacles to rapid development of an effective vaccine against pandemic influenza.
